# Reliability and quality of information provided by artificial intelligence chatbots on post-contrast acute kidney injury: an evaluation of diagnostic, preventive, and treatment guidance

**DOI:** 10.1590/1806-9282.20240891

**Published:** 2024-12-02

**Authors:** Seray Gizem Gur Ozcan, Merve Erkan

**Affiliations:** 1 Bursa Yuksek Ihtisas Education and Research Hospital, Department of Radiology – Bursa, Türkiye.; 2 Bursa City Hospital, Department of Radiology – Bursa, Türkiye.

**Keywords:** Artificial intelligence, Contrast media, Acute kidney injury

## Abstract

**OBJECTIVE:**

The aim of this study was to evaluate the reliability and quality of information provided by artificial intelligence chatbots regarding the diagnosis, preventive methods, and treatment of contrast-associated acute kidney injury, while also discussing their benefits and drawbacks.

**METHODS:**

The most frequently asked questions regarding contrast-associated acute kidney injury on Google Trends between January 2022 and January 2024 were posed to four artificial intelligence chatbots: ChatGPT, Gemini, Copilot, and Perplexity. The responses were evaluated based on the DISCERN score, the Patient Education Materials Assessment Tool for Printable Materials score, the Web Resource Rating scale, the Coleman-Liau index, and a Likert scale.

**RESULTS:**

As per the DISCERN score, the quality of information provided by Perplexity received a rating of "good", while the quality of information acquired by ChatGPT, Gemini, and Copilot was scored as "average." Based on the Coleman-Liau index, the readability of the responses was greater than 11 for all artificial intelligence chatbots, suggesting a high level of complexity requiring a university-level education. Similarly, the understandability and applicability scores on the Patient Education Materials Assessment Tool for Printable Materials and the Web Resource Rating scale were low for all artificial intelligence programs. In consideration of the Likert score, all artificial intelligence chatbots received favorable ratings.

**CONCLUSIONS:**

While patients increasingly utilize artificial intelligence chatbots to acquire information about contrast-associated acute kidney injury, the readability and understandability of the information provided may be low.

## INTRODUCTION

Contrast-associated acute kidney injury (CA-AKI) is defined as the deterioration of renal function within three days following the administration of intravascular iodinated contrast material, with no other identifiable cause playing a role in the etiology^1^. With the advancement of diagnostic and interventional techniques and the increasing use of contrast agents in radiology, CA-AKI has become a significant cause of iatrogenic acute kidney injury^1^. The incidence of CA-AKI is more than 2% in the general population^2^.

In a meta-analysis study conducted in 2019, which reviewed studies on CA-AKI from various countries, 23 studies from Turkey were observed. The study, which involved 17,945 individuals, found that CA-AKI developed in 2,571 patients (14.7%), and the mortality rate in patients with CA-AKI was 14.6%. In this meta-analysis, the CA-AKI rates were 14.7% in the United States, 12.4% in China, 14.5% in Japan, and 12.9% in Italy^3^.

The pathophysiology of CA-AKI involves vasoconstriction, oxidative stress, and hypoxia^4^. To prevent CA-AKI, some recommended methods include pre-hydration before contrast-enhanced imaging, the use of N-acetylcysteine, and minimizing the contrast dose to the lowest possible level^5^.

Artificial intelligence chatbots (AICs), deep learning, and digital health terms have been successfully integrated into daily practice^6^. An AIC is a broad computer technology that enables machines to perform tasks and interpretations that normally require human intelligence. Tasks such as natural language understanding, image recognition, decision-making, and problem-solving are encompassed within AICs^7^. Chatbots utilize natural language processing to respond to user queries by matching them with the pre-compiled optimal responses of the system^8^.

The recent launch of ChatGPT by OpenAI, which integrates deep learning and generative pre-training transformer architecture-based language models, has significantly enhanced the capabilities of chatbots. Information obtained online can be inconsistent, making it difficult to distinguish accurate information from misinformation^9,10^. Despite much of the information being commercially driven, patients may not discern this, and without appropriate guidance, internet-based information can be harmful, confusing, and misleading^9^.

The aim of this study was to evaluate the reliability and quality of information provided by AICs concerning the definition, preventive measures, and treatment of CA-AKI. Moreover, the study sought to address the benefits and drawbacks of using AICs from the users’ perspective in this context.

## METHODS

Between January 2022 and January 2024, the most frequently asked questions related to CA-AKI on Google Trends were posed to four AICs: ChatGPT, Gemini, Copilot, and Perplexity. These questions included "What is CA-AKI?," "What are the preventive methods for CA-AKI?," and "What treatments are used for CA-AKI?." The responses provided by the AICs were evaluated blindly by two different radiologists, and the DISCERN score was calculated separately. The results were evaluated by two radiologists, and a single result was reported. The responses regarding CA-AKI were evaluated in reference to the European Society of Urogenital Radiology guidelines updated in 2018^11^. DISCERN is a scoring-based questionnaire system that evaluates the quality of information provided to patients based on evidence and unbiased information. The DISCERN system consists of 16 questions scored on a scale of 1 to 5. The adequacy of the information is scored by the user based on the accuracy of the information. DISCERN scores are interpreted as follows: scores ranging from 16 to 26 points indicate poor quality, 27 to 38 points indicate low quality, 39 to 50 points indicate average quality, 51 to 62 points indicate good quality, and 63 to 75 points indicate excellent quality^12,13^.

The Patient Education Materials Assessment Tool for Printable Materials (PEMAT-P) is a reliable and valid assessment system for evaluating the understandability and actionability of information obtained from printed brochures, flyers, websites, and audio and visual materials, for both individuals without medical education and healthcare professionals. Scores obtained for understandability and actionability are presented in percentages^14^.

The Web Resource Rating (WRR) scale, scored from 0 to 100%, is used to measure the reliability and transparency of information obtained online^15^. The Coleman-Liau index is employed to measure readability, and scores above 11 points indicate that reading is very difficult and suggest that at least a university-level education is required^16^.

In this study, median values were utilized for the scores obtained from the scoring systems, number of recommended treatments, and consistency of treatment options, while mean values were used to present the word count and Coleman-Liau index results.

### Data availability

The data associated with the study are not publicly available but are available from the corresponding author on reasonable request.

## RESULTS

The most frequently asked three questions related to CA-AKI were posed to four AICs by two radiologists. The scores acquired were interpreted, and different scores were re-evaluated to obtain a unified score. The responses regarding CA-AKI were evaluated in reference to the European Society of Urogenital Radiology guidelines updated in 2018^17^.

Table 1 presents the data regarding the word count, Coleman-Liau index, PEMAT-P scores, WRR score, number of treatment options presented to the patient, and adherence to the guidelines. As per the Coleman-Liau index, the readability of the responses was above 11 in all AICs, indicating a complexity requiring a university-level education. The PEMAT-P understandability and actionability scores were low for all AICs. Similarly, the WRR scores for all AICs were low. Based on the Likert scale, the AICs performed very well in providing information about preventive and therapeutic procedures. Almost all the treatment options recommended by the guidelines were available in the AICs. Table 2 shows the DISCERN scores of the AICs, indicating the quality of information regarding disease identification and treatment.

**Table 1:** Evaluation of the results of readability, understandability, and reliability of the artificial intelligence chatbots.

	Gemini	Perplexity	ChatGPT	Copilot
Word count	380	347	644	474
Consistency of treatment options offered according to the guidelines (Likert 1–5)	5	3	4	3
Coleman-Liau index	16.1	19.98	20.12	18.68
WRR score	19.6	39.28	19.6	39.28
Number of recommended treatment alternatives	6	3	5	3
PEMAT-P understandability score	38.5	50	53	38.5
PEMAT-P actionability score	33.3	50	33.3	50

WRR: Web Resource Rating, PEMAT-P: Patient Education Materials Assessment Tool for Printable Materials.

**Table 2:** Median DISCERN scores of the artificial intelligence chatbots.

DISCERN question		Gemini	Perplexity	ChatGPT	Copilot
1	Are the aims clear?	4	5	5	4
2	Does it achieve its aims?	5	5	4	4
3	Is it relevant?	3	5	5	4
4	Is it clear what sources of information were used to compile the publication (other than the author or producer)?	3	4	1	4
5	Is it clear when the information used or reported in the publication was produced?	1	1	1	1
6	Is it balanced and unbiased?	4	5	4	5
7	Does it provide details of additional sources of support and information?	3	2	1	2
8	Does it refer to areas of uncertainty?	3	3	2	2
9	Does it describe how each treatment works?	2	4	4	3
10	Does it describe the benefits of each treatment?	3	4	4	5
11	Does it describe the risks of each treatment?	1	2	1	1
12	Does it describe what would happen if no treatment is used?	1	1	1	1
13	Does it describe how the treatment choices affect the overall quality of life?	1	1	1	1
14	Is it clear that there may be more than one possible treatment choice?	3	4	5	4
15	Does it provide support for shared decision-making?	4	3	4	3
16	Based on the answers to all of the above questions, rate the overall quality of the publication as a source of information about treatment choices.	3	4	3	4
	Total DISCERN score	44	53	46	48

All AICs provided sufficient responses regarding the definition of CA-AKI, listing underlying causes, identifying those more commonly affected, and determining risk factors. Since ChatGPT did not cite sources for the information provided, making it difficult to assess the reliability of the information, it did not receive a high score in this aspect. However, Copilot and Perplexity excelled, particularly in referencing, earning high scores in this regard. All AICs provided adequate information on preventive methods and measures to prevent CA-AKI. Only Gemini directed users to healthcare professionals in the presence of any doubts about potential risks.

According to the DISCERN score, Perplexity provided information of good quality with a total score of 53 regarding CA-AKI, while the quality of information provided by ChatGPT with a score of 46, Gemini with a score of 44, and Copilot with a score of 48 was "average." The content presented by Perplexity was more easily understandable due to its concise presentation of the necessary information with the least word count. As all AICs contained general information, they did not offer personalized or specific treatment options. Additionally, none of the AICs addressed potential problems if individuals did not receive treatment or difficulties and complications that may arise during treatment. According to the DISCERN scoring, Perplexity received the highest score, followed by Copilot, ChatGPT, and Gemini.

## DISCUSSION

Artificial intelligence (AI) was first used in medicine in the early 1950s^18^. AI has played a vital role worldwide in the last few decades^19^. Particularly, with the free usage of ChatGPT version 3.5, the use of AICs has become ubiquitous in daily life. Undoubtedly, the most significant area where AICs have rapidly grown is the field of medicine, where they have begun to be frequently used by patients to promptly access information, increasing their understanding of treatment methods and diagnoses pertaining to their conditions^20^.

Patients have long had a desire to obtain information about their diseases. A study conducted by Keten and Erkan revealed that patients’ use of YouTube videos for information regarding undescended testicles was very high; however, the reliability of the information was low, at around 26%^21^. YouTube videos rely entirely on the information provided by the content creator and are subjective.

In recent years, AI technologies have begun to compile internet-based medical information to provide more reliable and academic literature. Some AICs even support their academic information with references. AI systems provide training on a specific dataset to predict better outcomes and assist in solving complex problems with high reliability^22^. This has led to speculations being made regarding the potential replacement of healthcare professionals by AICs with the introduction of AI systems in the medical field^23^.

The growing population and longer life expectancy are increasing the need for healthcare professionals in society, which appears to make it impossible for professionals to meet every societal need. In this regard, the recommendation of the World Health Organization on "Self-Care Interventions for Health" stands out. Due to increasing health problems, it will not be possible for healthcare professionals to provide one-on-one care for every patient. In such cases, AICs will play an important role as assistants rather than replacing healthcare professionals^24^.

All AICs provided sufficient information on the definition of CA-AKI, listed the underlying causes in bullet points, identified the groups more frequently affected, and determined the risk factors. However, all AICs failed to provide adequate details on topics included in the DISCERN score, such as information on resources, possible outcomes if the patient declines treatment, impacts of treatment options on the patient's quality of life, and the risks of treatment. Consequently, they received relatively poor scores in the scoring system. Since the requests by patients to AICs are generally superficial, the responses are brief and concise without addressing every disease comprehensively. While the general information may be sufficient, they might fall short on specific topics, resulting in lower scores.

Copilot and Perplexity facilitated the referral of patients to relevant articles by providing information and referencing the respective sources. However, scholars in medicine and the humanities have long emphasized the value of personal narratives that provide perspective on patients’ lives. Numerous studies have been conducted on the value of listening to, reading, and writing patients’ disease narratives^25^.

Once patients acquire information about their diseases, they feel the need to learn about personalized treatment options in order to understand the potential challenges of treatment and be aware of the situations that they and their families may face during the treatment process. This indicates that while patients can obtain general information about their diseases from AICs, they still need healthcare professionals for more specific treatment options and recommendations.

In this study, the AICs provided sufficient information regarding CA-AKI and explained the underlying conditions according to the DISCERN score, categorizing the information by headings to facilitate understandability. However, all AICs fell short in personalizing information and addressing potential experiences during the treatment implementation process. Various cancer types rank at the top of search engine queries^26^. In diseases such as cancer that are stage-dependent, it is more challenging for AICs to provide personalized information. However, CA-AKI is a field independent of disease stages, containing more general information. As a result, the DISCERN scores were fair for Copilot, ChatGPT, and Gemini and good for Perplexity. The PEMAT-P scores, encompassing understandability and actionability, were high, indicating that the understandability and actionability of the information provided would be difficult for those without a university-level education.

In a urological study examining AICs conducted by Talyshinskii et al., AICs have become focal points in fields such as urological symptom control, health screening, patient education, counseling, lifestyle changes, conservative management, clinical decision support, and post-treatment follow-up care^27^. In a previous study by Sheikh et al. comprising questions about acute kidney failure and renal replacement therapies asked to AICs, ChatGPT generated responses with a high accuracy rate of 97% related to acute kidney failure. It demonstrated that ChatGPT 4.0 is consistent in interpreting and responding to acute kidney failure regardless of linguistic changes, and it has a high accuracy rate. Moreover, it emphasized that further research is necessary to investigate the direct impact of AI-generated responses on patient understanding and education outcomes^28^. In light of previous studies, AICs provide accurate and reliable information on acute kidney injury, but a sufficient educational background is required for understanding. Nevertheless, there is a lack of studies comparing this information across different AICs.

The essential limitation of this study is the subjective nature of scoring the information provided by the AICs, which may vary among evaluators. This limitation can be mitigated by having AICs themselves perform the scoring, potentially yielding more objective results, instead of relying on separate assessments by two radiologists.

## CONCLUSION

With increasing life expectancy and the growing significance of preventive medicine, the availability of radiological diagnostic methods has increased in contemporary medical practice. Patients utilize AICs to acquire information related to the potential CA-AKI associated with contrast agent use. However, the readability and understandability of the information require a university-level education for comprehension. Therefore, AICs require further development to cater to the needs of society and individuals without a medical background.
